# River Ecological Corridor: A Conceptual Framework and Review of the Spatial Management Scope

**DOI:** 10.3390/ijerph19137752

**Published:** 2022-06-24

**Authors:** Qi Han, Xiaogang Wang, Yun Li, Zhengxian Zhang

**Affiliations:** 1 College of Water Conservancy and Hydropower Engineering, Hohai University, Nanjing 210098, China; qhan@hhu.edu.cn; 2 State Key Laboratory of Hydrology Water Resources and Hydraulic Engineering, Nanjing Hydraulic Research Institute, Nanjing 210029, China; zhangzx@whu.edu.cn; 3 State Key Laboratory of Water Resources and Hydropower Engineering Science, Wuhan University, Wuhan 430072, China

**Keywords:** the river ecological corridor, territorial spatial control line, spatial management scope, minimum boundary, outermost boundary

## Abstract

Studying the spatial management scope of the river ecological corridor is a crucial step in effectively managing river health problems. For various purposes and needs, human beings intervene excessively in the river, resulting in the problems of unclear spatial scope, unclear ownership, and unreasonable functional utilization of the river ecological corridor. However, there is scarce research on the management scope of the river ecological corridor at present, and on the coordination relationship with territorial spatial protection planning. Therefore, in order to solve this key problem, this paper reviews and summarizes the current research status and development trends in terms of the concept, components, and other basic theories of the river ecological corridor, as well as relevant policy regulations. The relationship between the spatial scope of the river ecological corridor and the territorial spatial control line is analyzed, including the relationship with the river shoreline, aquatic ecological redline, “three control lines” and other control lines. Accordingly, this study reviewed the spatial management and control scope of the river ecological corridor. It also determined that the boundary line of the river shoreline management is the minimum line, the aquatic ecological redline, and the “three control lines” are the outermost boundary lines, in which the aquatic ecological redline has priority over other control lines. It also points out the thinking of determining the management scope in the protection and restoration of the river ecological corridor in the future. Our findings can provide a decision-making basis for the management of river ecological space.

## 1. Introduction

The management of river ecological corridor is an important measure to revive the ecological environment of the river and realize harmonious coexistence between humans and water. The early remediation and exploitation of rivers have changed the landscape pattern of natural rivers to a great extent. And the ecological space of rivers has been squeezed by activities such as the reclamation of farmland from lakes, agricultural production, and urban construction. Finally, the spatial scope of the river is unknown, the ownership of the river is unclear, and the development of the shoreline is disordered. In addition, the subsequent supervision is ineffective, resulting in the degradation of river shoreline ecology and function, and surface water ecological problems have become increasingly serious [1]. Excessive shipping and hydropower development projects lead to serious channelization of rivers, deterioration of water quality, destruction of riparian ecosystems, and the threat of flooding in river basins. Typical examples include the Kissimmee River in the United States [2], Rhine River in Europe [1], Isar River in Germany [3], Kushiro River in Japan [4], and Laohe River in China [5].

With the continuous strengthening of people’s awareness of river ecological space protection, various domestic and foreign laws and regulations have also been promulgated (Table 1). In 1997, the “Fluvial Law” of Japan put forward a series of river governance systems that integrate water control, water conservancy, and water environment, and the law has been used to this today [6,7,8]. In 2019, the U.S. Environmental Protection Agency (EPA) and the U.S. Army Corps of Engineers (USACE) defined federally regulated waters under the Clean Water Act (CWA) and suggested that the adverse effects on the ecological environment and biological communities should be minimized when constructing and using hydraulic engineering [9]. In December 2021, the “14th Five-Year Plan for Water Security Guarantee” [10] and “Implementation plan for restoring the ecological environment of rivers during the 14th Five-Year Plan period” [11] were issued by China. It proposes the promotion, protection, and restoration of river shorelines and the construction of the ecological corridor to clarify the management and control scope of the river. In March 2021, the “Yangtze River Protection Law” [12] implemented by China calls for strengthening the protection work of rivers in the Yangtze River Basin, delimiting the scope of river shorelines protection, and formulating protection plans to promote efficient use of river shorelines.

At present, the research on the determination of the river corridor boundary focuses on the theoretical aspect at home and abroad. The relevant research on the spatial scope of the river ecological corridor is mainly focused on the spatial structure. Internationally, the determination of the river corridor width is mainly studied for different protection purposes. For example, the research on the minimum width of the corridor for the purpose of biological protection or flood discharge demand, the research on the width of the corridor under river regulation and reconstruction, and the indirect research on the width of the buffer zone as the main content. For the purpose of biological protection, the determination of corridor protection width is mainly based on the empirical value determined by observation for specific species [19,20,21]. In the comprehensive management of rivers, some studies analyze the evolution of corridor by combining the reconstruction of corridor evolution trajectory with possible controlling factors, so as to determine the width of the river corridor [22,23]. The related research on the buffer width mainly applies mathematical models and numerical calculation methods [24,25,26].

**Figure 1:**
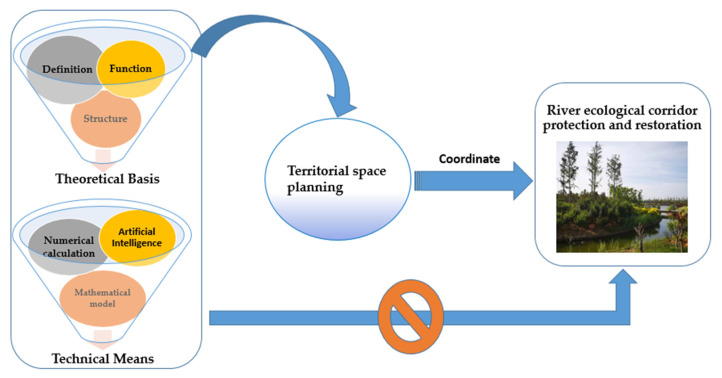
A diagram of the coordination relationship between the research on the scope definition of the river ecological corridor and the territorial spatial planning. The research on the scope definition of the river ecological corridor is carried out in combination with theoretical basis and technical means. It is necessary to coordinate with territorial spatial planning to effectively protect and restore the river ecological corridor.

Although various countries have issued various laws and regulations to limit human activities to a certain extent, the research and actual management work of river ecological space are still not in-depth and effective. Japan’s water management boundary is mostly based on local administration, national boundaries, and other boundaries. The continuous flow of water bodies in nature carries out segmented management. Although the management boundary is clear, how to manage “transboundary water” is a key issue. For another example, the United States has constantly revised the scope of waters under federal jurisdiction. In the 2020 navigable waters protection rules, it is proposed to solve the problem of the boundary of federally administered waters being accurately defined as soon as possible [9]. The European Union (EU) proposes to identify the watershed management area, formulate the watershed management plan and effectively manage the water environment [27]. However, China’s environmental protection of rivers is coordinated by multiple departments, such as the Ministry of Water Resources, the Ministry of Ecological Environment, the Ministry of Forestry, and the Bureau of Land and Resources. However, the coordination of the cross-sectoral river corridor protection boundary is not perfect at present. Comparing the current research on the management scope of river ecological corridor in various countries (Table 2), it is found that the problems of disordered development and utilization of river shoreline, “transboundary water” and multi-department coordination are caused by unclear boundary and ownership of the river ecological corridor is widespread. Hence, the step of territorial spatial coordination is indispensable for the protection and restoration of the river ecological corridor (Figure 1), which needs to be further clarified.

Through the analysis of the above research, it is obvious that the research on territorial regulation is not enough in many countries, and China is also lacking in this regard. What this study focuses on is the territorial space control line determined by various laws and regulations. How to coordinate the relationship among them? How to carry out the research on the management scope of the river ecological corridor space, so as to maximize the maintenance of the integrity and continuity? How to exert its ecosystem service function through the spatial scope management of the river ecological corridor? The core issue is to clarify the dominant function and ownership relationship of ecological space, to determine the boundary of the river ecological corridor. This study will discuss and analyze this point and put forward key ideas and methods. It can provide a targeted, critical and comprehensive theoretical basis for the decision-making of river ecological space management and control. It also can provide a scientific method and decision-making basis for the river ecological corridor management in China.

In the rest of this paper, Section 2 introduces the definition, structure, and function of the river ecological corridor in detail, to lay a theoretical foundation for the next work. Section 3 combs and analyzes the relationship between the river ecological corridor and land spatial management scope line. Section 4 explores the scope of the river ecological corridor based on territorial space management; Section 5 discusses the principles for defining the scope of the river ecological corridor and the limitations of this study. The conclusions are in Section 6.

## 2. Theoretical Concepts and Structure

### 2.1. Concept Discrimination

#### 2.1.1. Ecological Corridor

“Corridor” is derived from landscape ecology. It refers to a linear landscape unit that is different from the matrix on both sides [32]. “Ecological corridor” is derived from the concept of “corridor”. It refers to the spatial type of linear or banded landscape ecosystem, which has the functions of ecological services, such as protecting biodiversity, filtering pollutants, preventing soil erosion, regulating floods, and so on [33,34]. The American Conservation Management Association defines an ecological corridor as “a narrow strip of vegetation for wildlife that can promote the movement of biological factors between the two places” [34]. In landscape ecology, it is considered that the ecological corridor is for the protection of biodiversity and connecting with the provenance habitat. It must have sufficient width, and even the wider the better [35]. The ecological corridor emphasizes biological pathways, which can connect other patches, enable specific species to migrate between patches [36,37] and transmit biological information [38]. An ecological corridor is also considered to be a corridor that is conducive to the environment. This corridor does not necessarily have to serve human beings or have vegetation on both sides, but it must have a positive impact on the environment [39].

Similar concepts include riparian zone, river greenways, vegetation buffer zone, and so on. Among them, there are two typical views on the definition of a riparian zone. The first is that it is a transitional semi-terrestrial and semi-aquatic transitional area regularly affected by flooding, and its boundary extends from the edge of the water body to the edge of the highland [40,41,42]. The second considers the riparian zone as a three-dimensional area of water-land interaction whose boundary extends outward to the limit of inundation and up to the river canopy of edge vegetation [43,44]. The river greenway and vegetation buffer zone are considered the transition zone between the river and terrestrial ecosystem. The green vegetation zone distributed along the river has the characteristics of a biochemical cycle and eco-hydrological functions [25,45]. The key structures of the above-mentioned concepts are the water-land interaction area, with ecology as the leading function. Therefore, this paper mainly defines an ecological corridor, that is, a linear or banded corridor aiming at ecological services, and the boundary extends from the intersection of the constant water level and the shore to the transition zone at the edge of the highland.

#### 2.1.2. River Ecological Corridor

Since the 20th century, studies on the river corridor has been gradually paid attention at home and abroad. There are relatively complete theoretical achievements and relatively mature practical experience now. Functionally, Little [46] divides the corridor into an urban water system corridor, leisure greenway, ecological natural corridor, landscape corridor, and comprehensive greenway network. Forman first proposed the “patch-corridor-matrix” model in landscape ecology, which divides the corridor into the linear corridor, the ribbon corridor, and the river corridor in terms of shape [29]. Structurally and functionally, some scholars divided the corridor into green belt corridor, green road corridor, and green river corridor [47,48]. In terms of type, some scholars classified the corridor into river corridor, road corridor, mountain corridor, and forest corridor [49,50,51]. Therefore, the river corridor is considered a branch of the corridor.

There are different views on the definition of a river corridor. Some scholars agree with Professor Forman’s definition of river corridor from landscape ecology, including river channels, floodplains, and highland transition zones, which provide living space and passage for organisms [52,53,54,55]. Some scholars define the concept of river corridor from vegetation ecology, which mainly refers to the vegetation zone distributed along the river [26,56]. Some scholars define it from the influence of human factors and believe that the structure of river corridor built with water conservancy projects such as embankments, reservoirs, and sluices are mainly restricted by the scope of project management and water area protection [57,58].

With the development of economy and society, the concept of ecology has been paid more and more attention by all countries. The concept of river corridor began to be defined from the perspective of ecology. In October 2006, the Ministry of Land, Infrastructure, Transport, and Tourism of Japan proposed the project model of “multiple natural rivers”, which believed that the healthy river corridor should ensure the natural ecology, and at the same time, social service functions should be combined to create various river corridors [6]. In 2020, EPA and USACE [9] proposed that water areas not only include relatively permanent water bodies but also habitats such as wetlands, emphasizing ecological functions. Alwin Seifert [59], a German landscape architect, proposed near-natural river treatment, arguing that the treatment of natural rivers should achieve the benefits of the ecological landscape closest to nature while achieving engineering benefits.

This study summarizes various previous narratives and believes that river ecological corridor should be an extension of the river corridor, including the ecological corridor in structure. The corridor is composed of biological elements and habitat elements in the river system ecosystem as the core content is more emphasized. In a narrow sense, river ecological corridor should be linear or banded corridor with a certain continuity, width, and ecological service functions, consisting of the river channel, floodplain, and highland edge transition zone. In a broad sense, river ecological corridor also include lakes, reservoirs, swamps, wetlands, natural embankments, estuarine areas, and other patchy areas.

### 2.2. Structure and Function

#### 2.2.1. Structure and Composition

**Figure 2:**
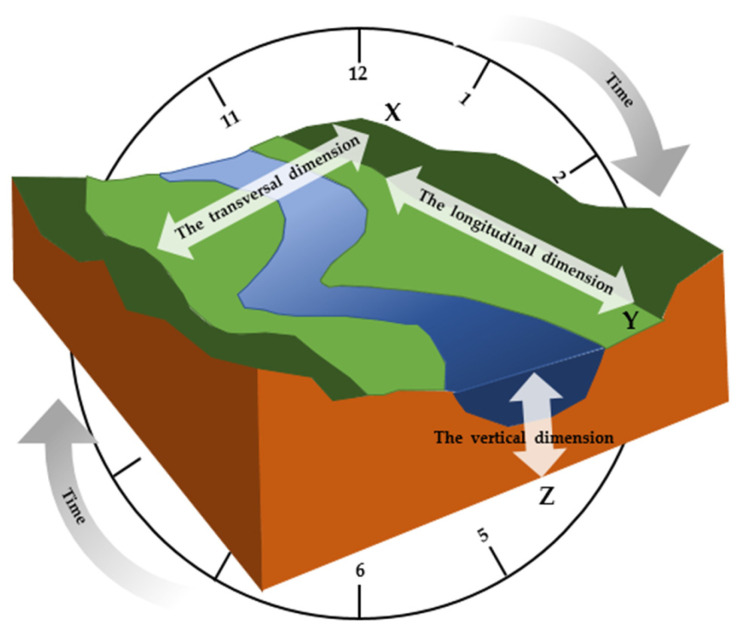
Four-dimensional structure diagram of river ecological corridor [52] (Reproduced with permission from [Dong Z.R.], [Eco-hydraulic Engineering]; published by [China Water Power Press], (2019)). The river ecological corridor includes the transversal dimension, the longitudinal dimension, the vertical dimension, and the time dimension.

In terms of river ecology, the composition of river ecological corridor has significant four-dimensional characteristics (Figure 2), namely the spatial dimensions of transversal, longitudinal, vertical, and temporal dimensions [52,60,61]. In terms of landscape ecology, the research on the specific structure of river ecological corridor includes the structure, curvature, width, and connectivity of the corridor [34,62].

**Figure 3:**
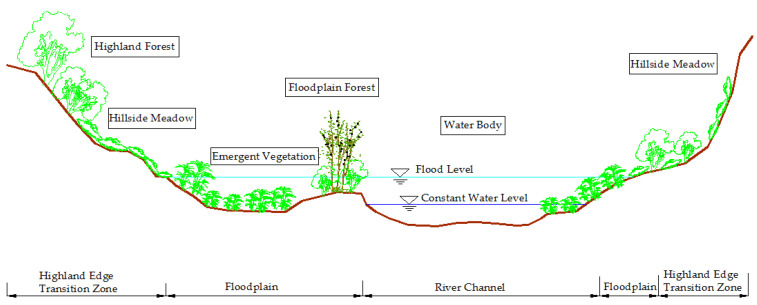
Cross-sectional structure diagram of the river channel. The transversal structure of river ecological corridor includes river channels, floodplain, and highland edge transition zone. The floodplain is mainly affected by the water level, which is the area of highly variable. The distribution of vegetation varies with the structure of the river ecological corridor, such as highland forest, emergent vegetation, floodplain forest, and hillside grassland.

The transversal dimension includes a river channel, floodplain, and highland edge transition zone with typical spatiotemporal characteristics (Figure 3). These areas have different spatial ranges during the long-term evolution of rivers. The floodplain is located on both sides or one side of the river channel and changes with flood inundation. The transition zone at the edge of the highland is the transition zone between the floodplain and the surrounding terrace [63,64,65]. In the longitudinal dimension, there are different hydrological and geomorphic characteristics from the river source, upstream to downstream and estuary. It is a continuous change gradient, which constitutes the physical and chemical process and biological community function of the river. It also shows continuity in the geographical space and dynamic processes [52,66]. In the vertical dimension, surface water, soil water, and groundwater are continuously alternately replenished to form a hydraulic connection. Therefore, the natural river corridor also includes shallow groundwater recharge areas, spring holes, and so on [67]. In the temporal dimension, river morphology and buffer zone biomes are also in dynamic succession. The spatial range, location, type, and flow of water habitat are all related to time. During the dry season, the riverside habitat shrinks, and the habitat changes in the interlaced water-land zone, which is directly related to the survival and reproduction of organisms [68].

#### 2.2.2. Function

**Figure 4:**
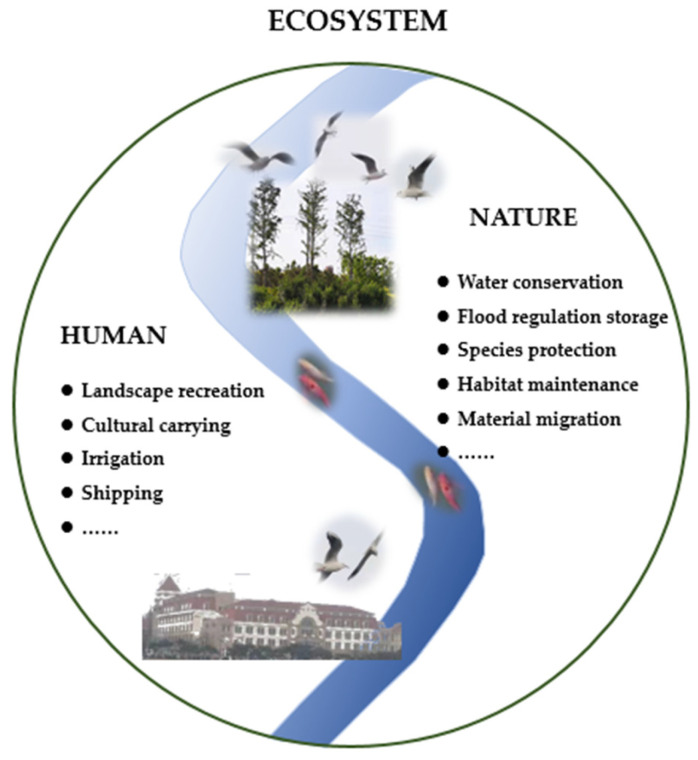
Ecosystem function diagram. Harmonious coexistence between humans and nature can jointly create a good ecosystem. Humans and nature have different functions for the river ecological corridor, including natural ecological function and social service function.

Rivers are the center of the breeding and development of human civilization. The river ecological corridor has both natural ecological functions and social service functions (Figure 4).

Natural ecological functions are manifested in water conservation, flood regulation and storage, soil and water conservation, species protection, habitat maintenance, material migration, and so on [41,54,55,69]. The main river channel is mainly a place for the exchange of water and sediment, a movement channel for fish and other organisms, and a place for aquatic vegetation to survive. It mainly plays the role of water purification and ensuring biodiversity. Floodplain is an area of flood inundation and ebb, with a large number of biological habitats. It is the zone with the richest species diversity. It mainly plays the functions of flood regulation, species protection, and habitat maintenance. The transition zone at the edge of the highland usually has dense vegetation, which plays a major role in controlling the inflow of sediments and nutrient components in the lateral watershed, controlling soil erosion and water conservation. As a corridor for wildlife, it can reduce the degree of habitat fragmentation.

Social service functions mainly include landscape recreation, cultural carrying, irrigation and shipping, and so on [70,71,72]. The river ecological corridor is linear open spaces composed of water flow, organisms, vegetation, and other elements. It is a natural landscape area. It can not only play the role of a biological channel and provide the resources needed by the city but also provide a place for human leisure, entertainment, and cultural inheritance. At the same time, it also provides convenience for the living needs of human irrigation and shipping.

Different functions endow the river corridor with different connotations and structural requirements. The dominant function is the first consideration in the definition of corridor scope. Different functions have different structural requirements, which need to reflect the structural characteristics of harmonious coexistence between man and nature.

## 3. The Relationship between River Ecological Corridor and Territorial Space Management and Control

It is necessary to clarify the ownership relationship between river ecological space and territorial space. In this way, space resources can be reasonably regulated, the function of the river ecological corridor can be brought into full play, and the ecological and economic benefits can be maximized.

### 3.1. River Ecological Corridor and River Shoreline

The development of human society has endowed the river corridor with a new connotation. The core content of current river ecological space management and control is “the management of river shoreline ”. In the “Dictionary of Geography” [73], “shoreline” is the concept of a line, which constantly changes with the ebb and rise of water. When it changes, it will extend to a certain range to the land, which indicates that the river shoreline is a banded area [74]. In the “Urban Water System Planning Specification” (GB50513) [75] issued in 2016, the shoreline is defined as the general term for the intersection of water and land, which is generally the range between the highest water level line and the constant water level line. In March 2019, the “Guidelines for Planning of River and Lake Shoreline Protection and Utilization (Trial)” issued by the Ministry of Water Resources [28] defined the shoreline as the strip area. This area is considered to be the interface between water and land, its range is between the waterfront control line and the outermost boundary line. From the perspective of water management, Zhang [76] defined the river shoreline as a certain range of banded areas at the water land junction. Among them, the area of the river refers to the water surface areas of rivers under the designed flood level or the highest historical flood level. The banded area refers to the space occupied by the river shoreline extending from the land to the water area. The size of this area is generally affected by the location of the river and the utilization of the shoreline.

**Figure 5:**
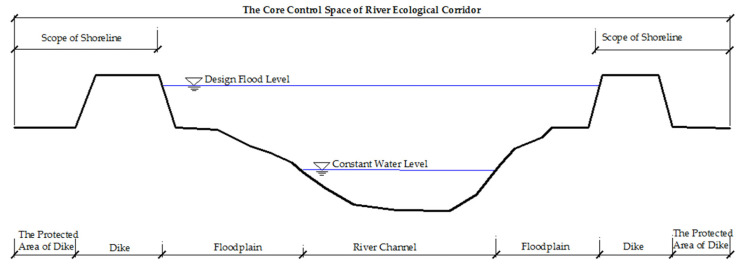
There is the core control scope of the embankment section. This section has a clear project management scope due to the relationship of the embankment. It is used to limit human activities and protect the ecological status of the river corridor.

**Figure 6:**
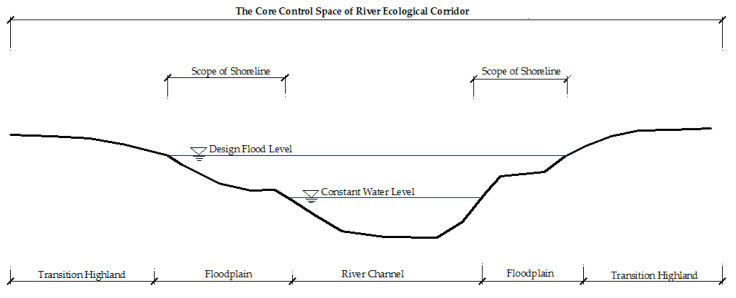
The core control area of the embankment-free section. In this section of no embankment, the width of the transition zone at the edge of the highland needs to be set on the basis of the shoreline, in order to control the negative impact of human activities on the river ecological corridor.

The river shoreline is a strip area, which not only has the natural ecological function attributes of flood discharge, water flow regulation, and maintenance of river health but also has the resource function attribute of development and utilization value under certain circumstances. When there is no embankment, the river shoreline range is between the normal water level and the design flood level (or the highest flood level in history), which belongs to the floodplain range in the composition of the river ecological corridor structure. It is considered the sum of the river shoreline range and the width of the transition zone at the edge of the highland. It is the core management space of the river ecological corridor (Figure 5). When there is a dike, the river shoreline range is determined by the dike protection management scope. From the perspective of structure, it belongs to the floodplain and highland edge transition zone in the structural composition of the river ecological corridor. However, the construction of embankments, revetment, and other projects will block the river’s ecological corridor horizontally. Therefore, at this time, the embankment management scope is the core control space (Figure 6).

### 3.2. River Ecological Corridor and “Three Control Lines”

The “three control lines” are mentioned in the “Guidance on the Unified Planning and Implementation of ‘Three Control Lines’ in Land and Spatial Planning” [29] issued by China. The “three control lines” are: (a) Ecological protection redline refers to the area with special important ecological functions that must be protected strictly. It is the bottom line and lifeline to ensure and maintain national ecological security. (b) Permanent basic farmland is cultivated land under special protection to ensure national food security and the supply of important agricultural products. (c) The urban development boundary is a regional boundary that can focus on urban development and functional utilization. In January 2017, the “Several Opinions on Delimiting and Strictly Abiding by the Ecological Protection Redline” [77] issued by China clarifies the definition of aquatic ecological redline. It is the boundary and bottom line for the protection scope of water ecological space based on the ecological protection redline [78].

In the development and utilization of land space, the ecological protection redline is the bottom line in the field of ecological environmental protection, and it is the key reference line for the management and control of river ecological space. Its priority is higher than the permanent basic farmland line and the boundary line of urban development [79]. The water ecological redline is the key line of the ecological protection redline [80], and its priority is also higher than the other lines. There are many kinds of areas with important ecological value including forests, lakes, drinking water source protection areas, swamps, and wetlands within the scope of the river ecological corridor that shall belong to the aquatic ecological redline. As a banded ecosystem, the river ecological corridor has different river sections passing through different protection and utilization areas. The scope of the aquatic ecological redline should be the area within the corridor that needs to be strictly protected. When the river ecological corridor is adjacent to the permanent basic farmland control line and the urban development boundary line, the ecological elements shall be considered first. The permanent basic farmland in the core protection area shall be withdrawn orderly, and those that are not in the core area and do not affect the ecological function can be retained. The ecological space within the boundary of urban development shall be given priority protection [81].

### 3.3. River Ecological Corridor and Other Scope Lines

Other scope lines in territorial spatial planning play a certain reference role in the delineation of the river ecological corridor. In the “Administrative Measures for Urban Blue Line” [30] and “Administrative Measures for Urban Green Line” [82], it is determined that the urban blue line is the geographical boundary of the main surface water body that needs to be protected and controlled within the urban planning area, and the urban green line is the range line of various types of urban green spaces such as protected green space and large-scale public green space. The riparian ecological blue line is expanded on the basis of the urban blue line, which has the same function as the urban blue line, except that it is aimed at other non-urban river channels and other areas without clear regulations [83,84]. The ecological health of the corridor is inseparable from these two lines. The blue line is the “blood line” of the river ecological corridor, which is structurally included in the scope of the river shoreline. Therefore, it is only used as a reference when delimiting the spatial scope of the river ecological corridor. The green line is the “epidemic prevention line” of the river ecological corridor and its scope is much larger than the river shoreline. It is more aimed at the urban landscape planning and from the perspective of river ecological protection and spatial control, its contribution is smaller than that of territorial spatial control lines such as the water ecological redline, but it still needs to be used as a reference line when necessary. The relationship between other scope lines and the river ecological corridor scope is shown in Table 3.

## 4. The Scope of River Ecological Corridor Based on Territorial Space Control

The appropriate spatial scope of the river ecological corridor plays an important role in the maintenance of the ecological environment, the protection of biodiversity, and the development of the social economy. It is an important step in river protection and restoration. On the basis that the relationship between the river ecological corridor and territorial spatial control lines has been clarified, further exploration into the delimiting of management and control scope of the river ecological corridor is required.

### 4.1. Spatial Management and Control of the River Ecological Corridor Based on Shoreline

Due to its special geographical location in the water-land interaction zone, the shoreline is an important part of the territorial space and a key area to maintain the ecological health of the river [85,86]. Since the 1950s, a series of the river corridor protection actions have been carried out at home and abroad. In terms of content, current research mainly focuses on ecological restoration riparian, zoning and classification of shoreline, evaluation of river resources, development potential evaluation of river shoreline, and technology of shoreline utilization and protection [87,88]. Technically, with the development of geographical information technology, the means of data collection, spatial analysis, and simulation prediction in 3S technology are gradually maturing [89]. This can quantify and visualize the results of research on spatial structure and pattern succession of the corridor at different scales.

**Figure 7:**
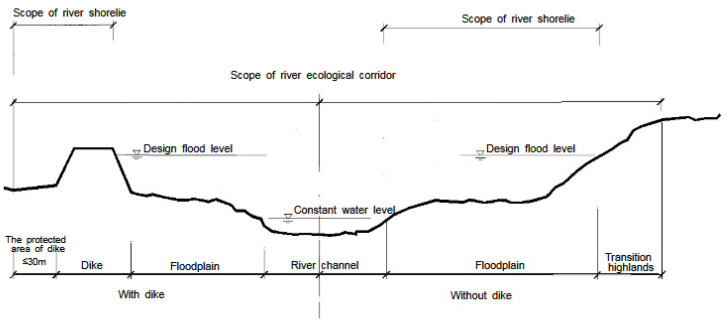
Schematic diagram of the river ecological corridor scope based on river shoreline. The scope of the shoreline is divided into two situations: with embankment section and without embankment section. Different situations lead to different ranges of the river ecological corridor. In the embankment section, the scope of the river ecological corridor is defined by the scope of the protected area of the dike. There is no dike section, its range is determined by the outer edge line of the transition zone at the edge of the highland.

In terms of the spatial management and control of the river ecological corridor based on the shoreline, most studies are carried out from three levels of basic theory, key technology, and strategic layout [90,91,92]. Theoretically, the relevant principles of landscape, ecology, and social economics are utilized. According to the longitudinal and transversal spatial characteristics of river shoreline, the impact mechanism of different utilization modes of shoreline on the main functions such as flood control safety, water supply safety, ecological environment, and social services is explored. The corresponding shoreline resource evaluation method is established. Software models from disciplines such as mathematics, physics, geography, and ecology are used for reference, such as spatial autocorrelation analysis, spatial local interpolation, landscape spatial dynamic model, big data analysis, remote sensing aerial photography, and so on. Then according to the established shoreline resources evaluation method to determine the importance of shoreline resources [85,93]. In terms of strategic layout, according to the importance of the assessed river shoreline resources and the relationship between the river ecological corridor and shoreline, appropriate methods should be adopted and deployed. In addition, the rational determination of the scope of the river ecological corridor should focus on the construction of dikes. In the section of the embankment, the minimum boundary line of the river ecological corridor is determined by the management and protection scope of the shoreline. In the section without embankment, according to the importance of shoreline resources and local leading functions, the reasonable determination of the transition zone at the edge of the highland needs to be considered in terms of flood control safety, water supply safety, ecological environment, and social services. That is, the outermost boundary line of the highland edge transition zone is the minimum boundary line of the river ecological corridor (Figure 7).

### 4.2. Spatial Management and Control of the River Ecological Corridor Based on Aquatic Ecological Redline

In “Several Opinions on Demarcating and Strictly Observing the Redline of Ecological Protection”, it is pointed out that ecological space is a territorial space with the main function of providing ecological products or services, covering all kinds of natural ecosystems in land and water areas [94]. The water ecological space is an ecological space that provides a place for the ecological-hydrological process, maintains the health and stability of water ecosystem, and ensures water security, including water space, land space for water conservation, and regional scope involved in flood discharge and storage [95]. The ecological protection redline and aquatic ecological redline are included in the ecological space, playing a bottom-line role in the management and control scope of the river ecological corridor. Internationally, there are many systems similar to the “ecological protection redline” in terms of ecological space management and control. For example, systems of natural protected areas, networks of compensative areas as an ecological infrastructure, ecosystem services, and national parks [96,97,98,99,100], which is the research and practice of the river corridor ecological protection and ecological security pattern construction.

At present, the research ideas for the spatial delineation of the river ecological corridor based on aquatic ecological redline are generally divided into two types, including classification and delimitation based on ecological space division [101,102,103,104] and identification based on key ecological elements [105,106]. The first idea starts from the perspective of the whole region. According to the degree of ecological importance and sensitivity, the ecological space of the whole area is divided. According to the type of natural attributes and dominant functions within the partition, the ecological space is classified. The ecological space management and control system oriented by classification and partition is constructed. The requirements for ecological space management and control are clarified, and the aquatic ecological redline is determined. In the water ecological space takes the water ecological redline as the outermost boundary line of the range, that is, the maximum boundary line. The second idea starts from the element unit, the importance of the types of protection elements is assessed in the river ecological corridor, and the particularly important ecological elements are controlled. The aquatic ecological redline is determined, and take this line is the boundary line of the outer edge of the river ecological corridor.

At present, the demarcation of permanent basic farmland control line and urban development boundary line has been relatively mature in China, which are the control lines in the field of land and urban development respectively. However, while ensuring the development of other fields, the ecology of the river corridor also should be ensured. In areas not covered by ecological protection redline or aquatic ecological redline, the permanent basic farmland line and urban development boundary line are considered as the maximum outer edge boundary line of the river ecological corridor. Therefore, the bottom line of the river ecological corridor should be the aquatic ecological redline, the permanent basic farmland control line, and the boundary line of urban development.

**Figure 8:**
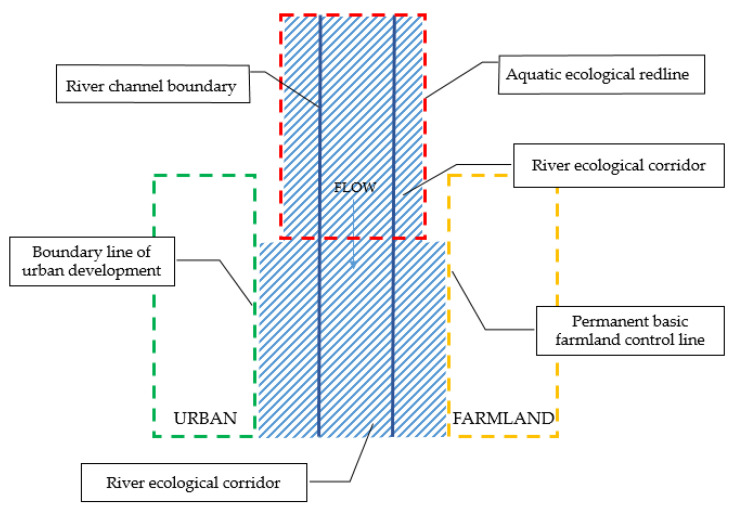
Schematic diagram of the river ecological corridor scope based on “three control lines”. The shaded part in the figure is the final scope of the river ecological corridor. The blue solid line is the boundary line of the main channel of the river, the red dotted line is the aquatic ecological redline, the green dotted line is the urban development boundary line, and the yellow dotted line is the permanent basic farmland control line. The aquatic ecological redline, boundary line of urban development, and permanent basic farmland control line can be used as the outermost boundary of the river ecological corridor. The aquatic ecological redline has a higher priority. When the aquatic ecological redline as shown in the figure overlaps with the urban development boundary line and permanent basic farmland line, the aquatic ecological redline shall prevail.

The relationship between the river ecological corridor and “three control lines” is shown in Figure 8.

## 5. Discussion

The previous paper combed the relationship between the river ecological corridor and territorial space. Combined with the territorial spatial control line, the scope of the river ecological corridor is defined. On this basis, this section discusses the principles and basic ideas of defining the spatial management scope of the river ecological corridor and puts forward the prospects and limitations of the research.

### 5.1. Principles for Defining the Scope of Space Management and Control

(a) The principle of consistency with dominant function and restoration goals.

The fundamental purpose of exploring the spatial management and control scope of the river ecological corridor is the protection and restoration of the river ecosystem. The determination of its dominant function and restoration goal is the primary principle. Different river sections have different functions and different goals, so as to different considerations for delimiting the scope. For example, in the river section with flood prevention as the main function, the flood overflow range is mainly considered. At this time, the boundary line of the flood plain is mainly concerned. For the river section dominated by ecological functions, the aspects of protecting biodiversity, ensuring water quality, and habitat maintenance are mainly considered. At this time, the width of the floodplain and the transition zone at the edge of the highland should be the primary consideration. For the river section with landscape culture as the dominant factor, the perspectives of hydrophilic platforms, proximity to nature, and entertainment are mainly considered, and the impact of human activities is also the focus factor. At this time, the width of the transition zone at the edge of the highland is the primary consideration.

(b) The principle of coordination with territorial spatial planning.

On the basis of considering the dominant functions and restoration objectives, the spatial scope of the river ecological corridor should be coordinated with territorial spatial planning. It includes the river shoreline, the permanent basic farmland control line, the ecological redline, the aquatic ecological redline, and the urban development boundary line. In the event of a conflict, various factors need to be considered for regulation.

(c) The principle of adaptation to local conditions.

The situation is different in different regions, and the original natural landscape characteristics and the construction planning of the existing project should be respected according to the actual situation. And it should be adapted to the local economic and social development. According to the requirements of different regions, the scope of the river ecological corridor should be reasonably determined.

### 5.2. Thinking on the Determination of the River Ecological Corridor Management and Control Scope

There are different degrees of construction and management of the river ecological corridor at home and abroad. In recent years, countries have paid more and more attention to the construction of the river ecological corridor. At present, China is also carrying out large-scale construction. The construction of the river ecological corridor is mainly carried out from the aspects of water security, water ecology, water environment, water resources, water landscape, and social economy. For example, the construction of the river ecological corridor in the Pearl River Delta region takes waterfowl as the key index and is carried out in a classified and hierarchical mode [107]. The construction of an ecological corridor in Shenzhen takes the land use and habitat sensitivity of the urban area as the key factors [108]. The construction of the ecological corridor, large rivers such as the Yellow River and the Yangtze River, takes the ecological environment status as the key index [109]. There is also a quantitative assessment of river health issues to characterize the construction status of the river ecological corridor [110,111]. Regarding the spatial coordination between the river ecological corridor and territory, some scholars have proposed that the territorial planning, ecological protection line regulations, and relevant planning standards should be used as policy tools for control in combination with the natural attributes of land and the land use conditions of the river ecological corridor [108]. In foreign countries, the United States, European countries such as France, the Netherlands, and Poland also focus on the river ecological corridor. For example, the greenway in the United States emphasizes the diversified construction of ecological environmental protection, landscape leisure and entertainment, and cultural preservation [112]. The conceptual framework of the European Ecological Network (EECONET) emphasizes the protection of the ecological environment and the network construction of landscape ecological connectivity [113,114]. Some scholars have proposed that the optimal management methods of the river ecological corridor use land-use change, riparian vegetation coverage, and natural conditions of the riverbank as key factors in the management of the scope of the river ecological corridor. The river ecological corridor is identified by means of GIS using the minimum cumulative resistance model [115,116]. However, specifically, the boundary of the management and control scope of the river ecological corridor has not been studied. The current research also fails to clarify its scientific basis. Due to the differences in natural landform, ecological environment, and other issues in various regions, the determination of the management and control scope of the river ecological corridor has become a key step.

**Figure 9:**
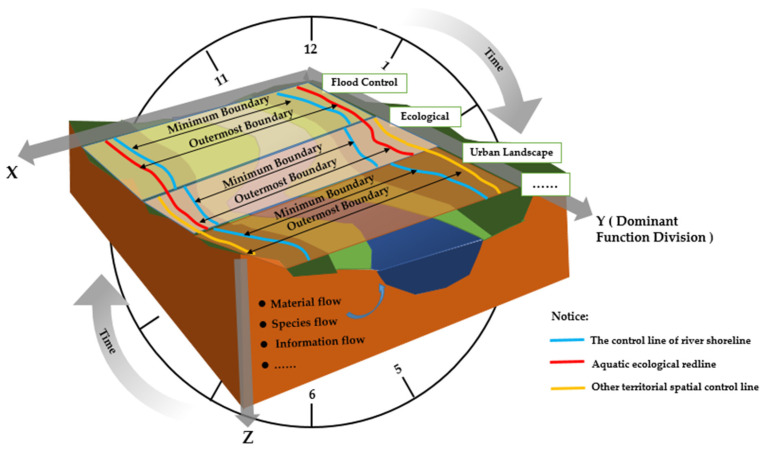
Schematic diagram of the river ecological corridor management scope. In the figure, the idea of the river corridor definition is illustrated with four dimensions. The river reaches with different functions lead to different the river ecological corridor scope. The lines in the figure are only used to illustrate different widths and do not represent the actual scope, which needs to be determined in combination with local conditions. Among them, the blue line represents the control line of the river shoreline, the redline represents the aquatic ecological redline, and the yellow line represents the other territorial spatial control lines (such as the boundary line of urban development, the control line of permanent basic farmland, urban blue line, the riparian ecological blue line, and urban green line).

On the basis of combing the research of the management scope of the river ecological corridor at home and abroad, it is concluded that the dynamic flow process under different times and spaces leads to the dynamic evolution of the corridor scope. This dynamic evolution is related to human beings. The combined action of social development activities has formed the geomorphological characteristics of the existing river ecological corridor. Based on this, this study puts forward the thinking of defining the scope of the river ecological corridor (Figure 9).

From the perspective of the overall scale, firstly, the leading functions and restoration objectives of the study area should be determined. Such as the functions of flood control and drainage, ecological function, and landscape culture as the leading factors. Based on the analysis of the longitudinal structure, the shoreline functional area and water ecological protection core area where different river sections are located is combined. The leading functions area of the corridor is divided on a longitudinal structure. 

From the corridor scale, which is also the most important scale, the transversal structure and the time dimension should be analyzed. The boundary positions of the main river channel, floodplain, and height edge transition zone are determined in different periods, such as flood period, flat water period, and dry period. According to whether the shoreline has an embankment or not, the minimum boundary line of the corridor should be preliminarily delineated. Then the relationship between the boundary line of its scope and the territorial spatial management and control line should be coordinated, such as the river shoreline, ecological redline, aquatic ecological redline, permanent basic farmland control line, urban development boundary line, blue line, and green line. The scope is further revised to determine the maximum boundary line of the river ecological corridor.

Finally, from the river section scale, the vertical structure should be analyzed. From the perspectives of material flow, species flow, and information flow, the impact of ecological factors on the transversal and longitudinal structure should be considered. According to the planning requirements of the region, the scope of the river ecological corridor should be reasonably determined.

### 5.3. Limitations and Future Research

As a key area of territorial space, the river ecological corridor has become a necessary spatial link to connect the life community of mountains, rivers, forests, fields, lakes, and grasses. The scope of the river ecological corridor is the main space to realize the ecological health of rivers. It is the main area for the protection and restoration of the river ecological corridor. In terms of supervision, it mainly depends on strengthening the management and control of ecological spaces such as river shorelines. Therefore, the relationship between river ecological spatial ownership and territorial spatial ownership should be clarified. The scope boundary of the river ecological corridor should be determined. At the same time, the ecological spatial pattern should be optimized to maximize the comprehensive value of each ecological space. It provides the decision-making basis for further management of river ecological space in the future and provides theoretical and technical support for river ecological protection and restoration. Although this study plays a certain reference role in the spatial management and control of the river ecological corridor, it still has shortcomings:

(a) As an important vegetation buffer zone, wildlife migration zone, biodiversity protection zone, and also as the minimum boundary in the delineation of the river ecological corridor, the transition zone of the highland edge has important research significance. Its reasonable width range needs to be studied in depth with scientific, comprehensive, and specific methods.
(b) For the delineation of the ecological protection redline and aquatic ecological redline, feasible management measures and scientific demarcation methods are very necessary. The current research is not in-depth, and the delineation needs further research and exploration to truly ensure the sustainable service function of the water ecosystem.

## 6. Conclusions

The construction of an ecological corridor is an important part of the construction of ecological civilization. For the management and control of ecological space, the river system is the core element, and the construction of the river ecological corridor is the core task. In order to promote the ecological restoration of the river corridor and realizes the construction of the river ecological corridor, this paper studies the spatial scope definition of the river ecological corridor and rationalized the management and control scope of ecological space. The research results mainly answer the following key questions, and the main conclusions are as follows:

(a) What are the river ecological corridor? What functions does it include?

This paper discriminates the research on the definition, structure, and function of the river ecological corridor at home and abroad. On this basis, the definition of the river ecological corridor is summarized from the narrow and broad aspects, which lays the foundation for the next step.

(b) What is the territorial spatial control line? What is the relationship between it and the river ecological corridor?

Compared to other studies, the relationship between the boundary line of the river ecological corridor and the control line of territorial space is clearly analyzed in this paper. It clarified that the positioning of the river ecological corridor spatial scope in the territorial spatial management and control. The river shoreline is the minimum boundary line of the river ecological corridor. The aquatic ecological redline, permanent basic farmland line and urban development boundary line are the outermost boundary lines of the river ecological corridor. And the aquatic ecological redline has a high priority. The urban blue line and urban green line shall be used as reference lines when necessary.

(c) How to define the spatial scope of the river ecological corridor? How can the research on the management and control scope of the river ecological corridor space be carried out scientifically and reasonably?

Firstly, the principle of defining the spatial management and control scope of the river ecological corridor is put forward. It is carried out with the main line of “functional positioning-spatial coordination-adjusting measures to local conditions”.

Secondly, the research on the definition of the management and control scope of the river ecological corridor based on territorial space has been discussed, including the ideas and methods of defining of the management and control scope in the two cases of river shoreline management and aquatic ecological redline.

Thirdly, the research on the spatial management and control scope of the river ecological corridor based on river shoreline and aquatic ecological redline is discussed, and the idea of determining the management and control scope of the river ecological corridor is proposed. From four dimensions (transversal, longitudinal, vertical, and temporal) and three scales (watershed, corridor, and river section), three defining principles are combined, the relationship between the river ecological corridor and territorial spatial is coordinated, and the minimum and maximum boundaries of the spatial management and control scope of the river ecological corridor is determined.

## Figures and Tables

**Table 1:** Relevant laws and regulations for river ecological protection at home and abroad.

Nationality	Release Time	Name	Remarks
Japan	1997	Fluvial Law	The system of water control, water conservancy, and water environment are integrated, and the territorial development plan is considered to ensure the comprehensive management of rivers [13].
	2014	The Basic Act on the Water Cycle	The management of river basins is carried out on a basin-by-basin and in a manner of comprehensive regional sovereignty. Efforts are made to restore the original appearance and the ecosystem [14].
USA	1972 (Amended in 1977)	The Clean Water Act	Tanking “restoring and maintaining chemistry, physics and biological integrity in national waters” is the legal goal [15].
	2019	Federal Register	Redefine the waters, terminate the jurisdiction of the “transient” rivers, and emphasize the impact of hydraulic engineering on the ecology [9].
China	2021	The 14th Five-Year Plan for Water Security Guarantee	The management of river and lake space should be strengthened to promote the protection and restoration of river shorelines, and the construction of the ecological corridor [10].
	2021	The Yangtze River Protection Law	The protection of the Yangtze River Basin must be strengthened, and the protection plan for river and lake shorelines must also be formulated [12].
Germany	2009 (Amended in 2013)	The General Provisions of Water Law	In order to protect water sources from adverse effects, water protection areas should be determined, flood areas should be protected, and natural landscapes should be maintained and regained [16].
	2018	Urban Nature Master Plan- Federal Government’s “Vibrant City” Action Plan	The water body and its flood plains are protected to maintain water quality and habitat. Creating a more tranquil and healthy environment to improve the quality of life [17].
European Union	2002 (Amended in 2014)	Water Framework Directive	Watershed management areas and plans should be determined to effectively manage the water environment [18].

**Table 2:** Research status and deficiencies of the river ecological corridor management scope in various countries.

Nationality/Department		Basis for Scope Definition	Angle of Consideration					Existing Problems
			Habitat Maintenance	Regulating Flood	Landscape Entertainment	Social Services	Territorial Regulation	
Foreign	Japan	Administrative boundary [14]	√	√	√	√		“Transboundary water” problem
	USA	Navigable waters [9]	√	√	√	√		The precise problem of boundaries
	European Union	Watershed management area [27]	√	√	√	√		The management of land and water
China	Ministry of Water Resources	River shoreline management scope [28]		√				Multisectoral coordination problem
	Bureau of Land and Resources	“Three control lines” [29]/Urban blue line [30]	√	√		√		
	Ministry of Ecological Environment	Ecological buffer zone [31]	√	√	√			

**Table 3:** Relationship between other scope lines and the river ecological corridor scope.

Related Concepts	Foundation	Relationship with the Scope of the River Ecological Corridor
Urban blue line	Administrative Measures for Urban Blue Line	The blue line is structurally included in the scope of the river shoreline and is the reference line when delimiting the scope of the river ecological corridor.
The riparian ecological blue line	Local laws and regulations	
Urban green line	Administrative Measures for Urban Green Line	In terms of structure, the green line is much larger than the river shoreline. It contributes less to the territorial spatial control line such as the aquatic ecological redline. But it still needs to be used as a reference line to delimit the scope of the river ecological corridor when necessary.
